# Identification of key modules and hub genes in glioblastoma multiforme based on co‐expression network analysis

**DOI:** 10.1002/2211-5463.13078

**Published:** 2021-02-09

**Authors:** Chun Li, Bangming Pu, Long Gu, Mingwei Zhang, Hongping Shen, Yuan Yuan, Lishang Liao

**Affiliations:** ^1^ GCP Center the Affiliated Traditional Chinese Medicine Hospital of Southwest Medical University Luzhou China; ^2^ Department of Hepatobiliary Surgery the Affiliated Traditional Chinese Medicine Hospital of Southwest Medical University Luzhou China; ^3^ Department of Emergency Medicine the Affiliated Hospital of Southwest Medical University Luzhou China; ^4^ Department of Neurosurgery the Affiliated Traditional Chinese Medicine Hospital of Southwest Medical University Luzhou China

**Keywords:** biomarkers, glioblastoma multiforme, survival, TCGA, WGCNA

## Abstract

Glioblastoma multiforme (GBM) is the most malignant primary tumour in the central nervous system, but the molecular mechanisms underlying its pathogenesis remain unclear. In this study, data set GSE50161 was used to construct a co‐expression network for weighted gene co‐expression network analysis. Two modules (dubbed brown and turquoise) were found to have the strongest correlation with GBM. Functional enrichment analysis indicated that the brown module was involved in the cell cycle, DNA replication, and pyrimidine metabolism. The turquoise module was primarily related to circadian rhythm entrainment, glutamatergic synapses, and axonal guidance. Hub genes were screened by survival analysis using The Cancer Genome Atlas and Human Protein Atlas databases and further tested using the GSE4290 and Gene Expression Profiling Interactive Analysis databases. The eight hub genes (*NUSAP1*, *SHCBP1*, *KNL1*, *SULT4A1*, *SLC12A5*, *NUF2*, *NAPB*, and *GARNL3*) were verified at both the transcriptional and translational levels, and these gene expression levels were significant based on the World Health Organization classification system. These hub genes may be potential biomarkers and therapeutic targets for the accurate diagnosis and management of GBM.

## Graphical abstract

The molecular mechanisms underlying the pathogenesis of glioblastoma multiforme remain unclear. In this study, data set GSE50161 was used to construct a co‐expression network for analysis. We found the two modules (dubbed brown and turquoise) and eight hub genes (*NUSAP1*, *SHCBP1*, *KNL1*, *SULT4A1*, *SLC12A5*, *NUF2*, *NAPB* and *GARNL3* that were most strongly associated with GBM. These hub genes may be potential biomarkers and therapeutic targets for the accurate diagnosis and management of GBM.

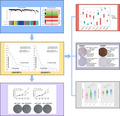

**Abbreviations**
AGGF1: angiogenic factors with G‐patch and FHA domain1
BPs: biological processes
CGGA: Chinese Glioma Genome Atlas
CIN: chromosomal instability
DEGs: differentially expressed genes
GBM: glioblastoma multiforme
GO: Gene Ontology
HPA: the Human Protein Atlas
IHC: immunohistochemically
KEGG: Kyoto Encyclopedia of Genes and Genomes
NDC80: the nuclear division cycle 80
SAC: the spindle assembly checkpoint
TCGA: The Cancer Genome Atlas
TFs: transcription factors
WGCNA: weighted gene co‐expression network analysis
WHO: the World Health Organization

Glioblastoma multiforme (GBM), which accounts for 47.1% of primary malignant brain tumours [1], is classified as a grade IV glioma by the World Health Organization (WHO) and a highly lethal tumour [2]. GBM is considered incurable, with a 5‐year survival rate of only 5.5% [1]. The poor prognosis is also related to the high recurrence rate of tumours despite aggressive multimodality treatments, including maximal surgical resection, radiotherapy, and chemotherapy [3].

Molecular data on brain tumours have a significant impact on prognosis and clinical management [4]. In 2016, the WHO classification of tumours of the central nervous system improved the traditional diagnostic criteria—which was based only on histological properties—and incorporated molecular markers, by establishing the ‘ISN‐Haarlem’ consensus [5]. Specifically, an integrated diagnostic approach based on clinical, histopathological, and molecular data is required to more accurately distinguish glioma tumour subtypes and define prognosis in response to specific treatments. According to a recent study, glioblastoma patients may benefit from molecular targeted therapies [6]. Here, we report two modules using weighted gene co‐expression network analysis (WGCNA) [7], which might provide potential biomarkers and therapeutic targets for more accurate diagnosis and treatment of GBM.

WGCNA, using soft‐thresholding techniques to convert a gene co‐expression similarity measure into a network connection strength, can reconstruct gene co‐expression modules and summarise modules using module eigengene [8]. WGCNA can yield more robust results as compared to unweighted networks [7]. WGCNA is one of the most extensive methods of genomic analysis, and it has a high degree of superiority by focussing on a group of genes, rather than a single gene, to minimise bias. In addition, WGCNA does not require cut‐off criteria and can retrieve momentous information, which is otherwise omitted with other methods. WGCNA has been applied in various cancers, such as pancreatic cancer, breast cancer, and osteosarcoma [9, 10, 11]. To identify key biomarkers and to further understand the molecular mechanisms of GBM, we used WGCNA to analyse GBM from a new perspective in this study.

## Materials and methods

### Data information

Two microarray profiles, including GBM samples (GSE50161 and GSE4290), were downloaded from the National Center for Biotechnology Information Gene Expression Omnibus (https://www.ncbi.nlm.nih.gov/geo/) [12]. As for the GSE50161 data set, 34 paediatric GBM samples and 13 normal samples were selected for WGCNA [13]. As for the GSE4290 data set, 81 malignant glioma cell samples and 23 nontumour samples from epilepsy patients were selected for validation [14]. The two data sets above were both based on the platform of the Affymetrix Human Genome U133 Plus 2.0 Array.

### Construction of WGCNA

GBM and normal samples were ranked by the median absolute deviation from large to small, and we precalculated the power parameter of the top 5000 genes using the pickSoftThreshold function of WGCNA [8]. This function provided the appropriate soft‐thresholding power for network construction by calculating the scale‐free topology fit index for several powers. Adjacency was turned into topological overlap, which could measure the network connectivity of a gene defined as the sum of its adjacency with all other genes for the network generation. Modules were grouped with tightly connected genes, which had similar expression profiles, and then identified on the dendrogram using the Dynamic Tree Cut algorithm. The first principal component of a given module is defined as the module eigengene, which can be considered as a representative of the gene expression profile in a module. The dissimilarity of module eigengenes was calculated to choose a cutline to merge some modules.

### Identification of significant GBM modules

To find significant gene modules, we created module–trait relationships to detect the correlation between module eigengenes and the GBM trait. The log_10_ transformation of the *P*‐value was defined as the gene significance. The higher the absolute value of gene significance, the more biologically significant the gene [8]. Module significance was determined as the average absolute gene significance measure for all genes in a given module. The module membership measure is highly related to the intramodular connectivity. Highly connected intramodular hub genes tend to have high module membership values to the respective module. Generally, the module with the absolute module significance ranked first among all the selected modules was considered as the one most related to GBM traits and was called the key module.

### Functional enrichment analysis

In order to gain further insight into the function of genes in key modules, eigengenes in the key module were subjected to Gene Ontology (GO) enrichment analysis to identify the enrichment results of biological processes (BPs), cell components, and molecular functions using the clusterprofiler r package [15]. Kyoto Encyclopedia of Genes and Genomes (KEGG) pathway enrichment analyses of key modules were also performed [16]. Fisher's exact test based on hypergeometric distribution was used for statistical analysis. *P* < 0.05 was considered to have statistical significance and to achieve significant enrichment.

### Identification of hub genes in key modules

R function exportNetworkToCytoscape was used to export the key modules as networks in text format for cytoscape (ver3.6.1, https://cytoscape.org/) [17]. After importing the texts in cytoscape, we sorted the node degree of genes in the key modules and screened the top 30 genes in each module. The Cancer Genome Atlas (TCGA, http://cancergenome.nih.gov/) is a landmark cancer genomics programme that contains over 20 000 molecularly characterised primary cancer and matched normal samples spanning 33 cancer types [18]. To further screen hub genes among the key modules, we downloaded the GBM gene expression data from TCGA database for survival analysis (grouped by median expression level) of the top 30 genes in each module above. If there were no significant results of survival analysis by TCGA in a given module, we used the Human Protein Atlas (HPA, https://www.proteinatlas.org/) to screen candidate genes associated with survival in the module by searching for the best cut‐off expression value in TCGA. By limiting the sample size in the group, the grouping was not less than 20% of the total sample in the HPA, and genes with the lowest log‐rank *P*‐values (log‐rank *P* < 0.05) of survival analysis were selected [19]. The R package limma was applied to screen differentially expressed genes (DEGs) between GBM and nontumour samples in GSE50161 [20]. The corresponding *P*‐values of less than 0.01 and log |fold change (FC)| larger than 1.5 were selected as the cut‐off criteria for DEGs. DEGs associated with survival were considered to be hub genes in GBM tumorigenesis.

### Validation of hub genes

We performed hub genetic verification in two separate data sets from different sources. For data set GSE4290, the screening methods and cut‐off criteria for DEGs were the same as in GSE50161. Volcano plots and hierarchical clustering analyses were carried out using R packages ggplot2 and pheatmap, respectively. GO function enrichment and KEGG pathway analysis were also performed on DEGs. The differential expression levels of these hub genes were also verified in GEPIA (http://gepia.cancer‐pku.cn/) [21]. The GBM and normal data sources were from TCGA and the Genotype‐Tissue Expression project [22]. In addition, the HPA database provided immunohistochemically (IHC) stained specimens of the proteins of genes. It was used for verifying the translational levels of hub genes in normal and tumour tissues (including three normal samples and at least nine GBM samples).

### Hub gene expression distribution (WHO classification)

To further investigate the distribution of these eight key genes in the WHO classification, we used R to analyse the mRNAseq_693 data set in the Chinese Glioma Genome Atlas (CGGA, http://www.cgga.org.cn/) database. The mRNAseq_693 data set contained 188 GBM samples in WHO grade II, 255 samples in grade III and 249 samples in grade IV. Gene expression data were subjected to a *t*‐test after log2 (expression + 0.001) conversion. A *P*‐value of less than 0.05 was considered statistically significant.

### Prediction of transcription factors of hub genes

Identification of the transcription factors (TFs) that operate a perturbed gene network and detection of their target genes are instrumental steps in uncovering key insights into oncogenic programmes, including the discovery of therapeutic targets [23, 24, 25]. The iRegulon in cytoscape is a plugin to reverse engineer the transcriptional regulatory network underlying a co‐expressed gene set using cis‐regulatory sequence analysis [26]. iRegulon relies on the analysis of the regulatory sequences around each gene in the gene set to detect enriched TF motifs or ChIP‐seq peaks, using databases of nearly 10 000 TF motifs and 1000 ChIP‐seq data sets or ‘tracks’. Next, it associates enriched motifs and tracks with candidate TFs and determines the optimal subset of direct target genes. In order to explore the TFs of hub genes in key modules, we used the iRegulon plugin to predict the TFs of hub genes in the two modules. The screening conditions were normalised enrichment scores > 3.

### Validates the role of high expression of Hub gene by siRNA transfection knockdown method

Hub gene identification has confirmed the high expression of four Hub genes of *NUSAP1, SHCBP1, NUF2* and *KNL1* in tumours. In order to further study the role of these four genes in GBM, we used siRNA transfection knockdown method to knock down the mRNA expression of four Hub genes of *NUSAP1, SHCBP1, NUF2 and KNL1* in human astroblastoma cell line U87 (it was purchased from the Committee on Type Culture Collection of the Chinese Academy of Sciences. Shanghai, China) and then determined the level of mRNA of four Hub genes by RT‐PCR method (All primers are listed in Table 1); next, we analysed the variation of proliferation and cloning ability of U87 cells by CCK8 method and clone formation experiment, and finally, the role of four Hub genes was validated.

**Table 1:** The sequences of siRNA and RT‐qPCR primers.

Gene	Primer	Tm
*SAP1*‐F	TGGACCTCTAATGATGGGCAG	60
*SAP1*‐R	AGGCTTGTTCTTGCGAATCCC	60
*SHCBP1*‐F	GGAAGTGTATCCTGTTGAGGGA	60
*SHCBP1*‐R	ACCAGGTATTGTTCCATCCTGT	60
*NUF2*‐F	GGAAGGCTTCTTACCATTCAGC	60
*NUF2*‐R	GACTTGTCCGTTTTGCTTTTGG	60
*KNL1*‐F	CTTCACACCGAGGACTCAAGA	60
*KNL1*‐R	TTTGATGTGTAGAAGAGGCACTG	60
*GAPDH*‐F	GGAGCGAGATCCCTCCAAAAT	60
*GAPDH*‐R	GGCTGTTGTCATACTTCTCATGG	60

### Statistical analyses

Data were analysed by using ibm spss 21.0 software (IBM, Chicago, IL, USA) and are presented as the mean ± SD. Quantitative PCR results and clone formation were analysed by Student's *t*‐test, and CCK8 cell proliferation was analysed by one‐way analysis.

## Results

### WGCN construction and module selection

**Fig. 1:**
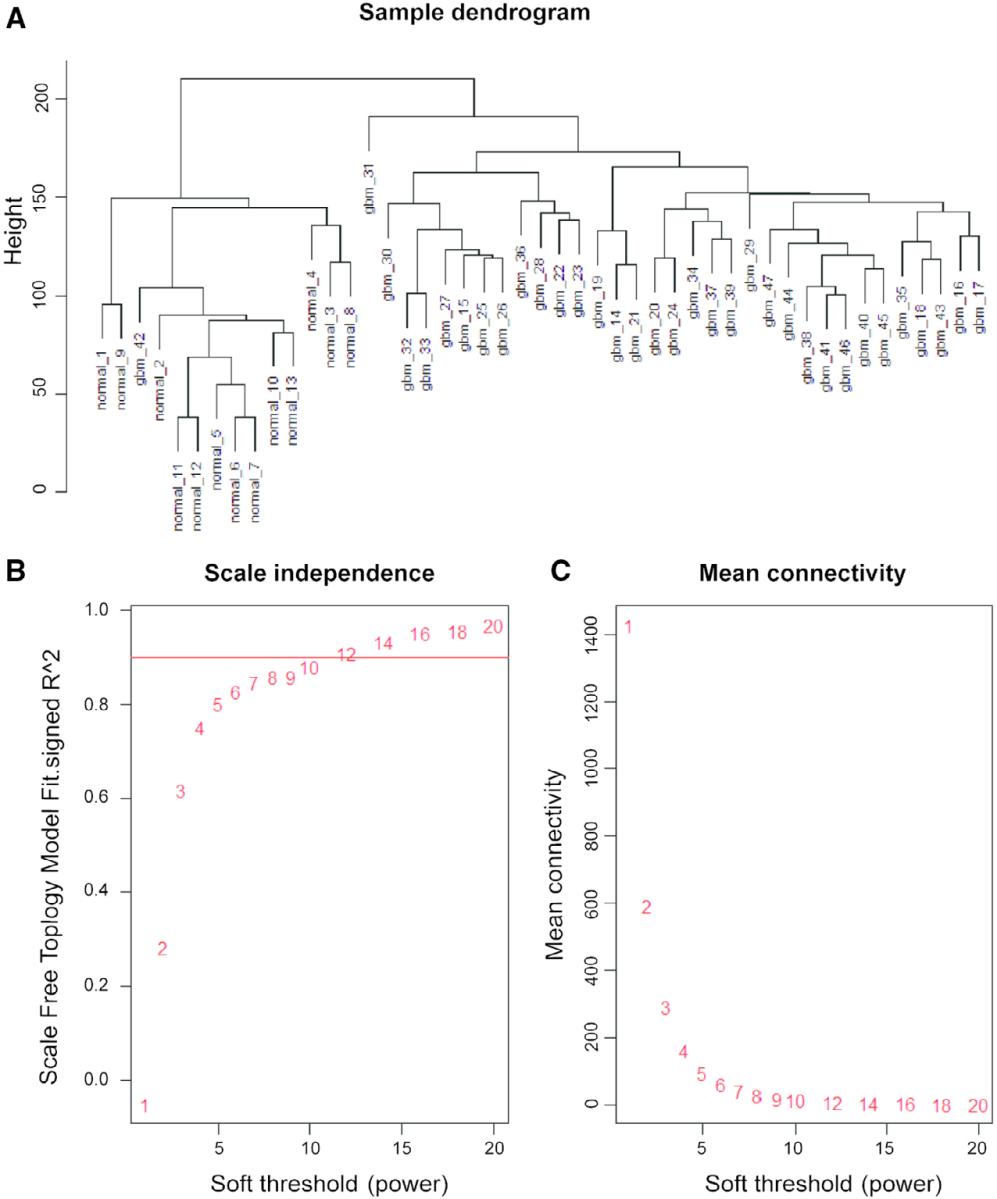
Clustering of samples and determination of soft‐thresholding power. (A) Clustering of samples. (B) The scale‐free topology fitting index (*R* ^2^, *y*‐axis) as a function of the soft‐thresholding power (*x*‐axis). The red line indicates *R* ^2^ = 0.9. (C) The mean connectivity (degree, *y*‐axis) is displayed as a function of the soft‐thresholding power (*x*‐axis). Red Arabic numbers in the panels denote different soft‐thresholds. β = 8, there was a trade‐off between maximising the scale‐free topology model fitting index (*R* ^2^) and maintaining a high mean number of connections.

**Fig. 2:**
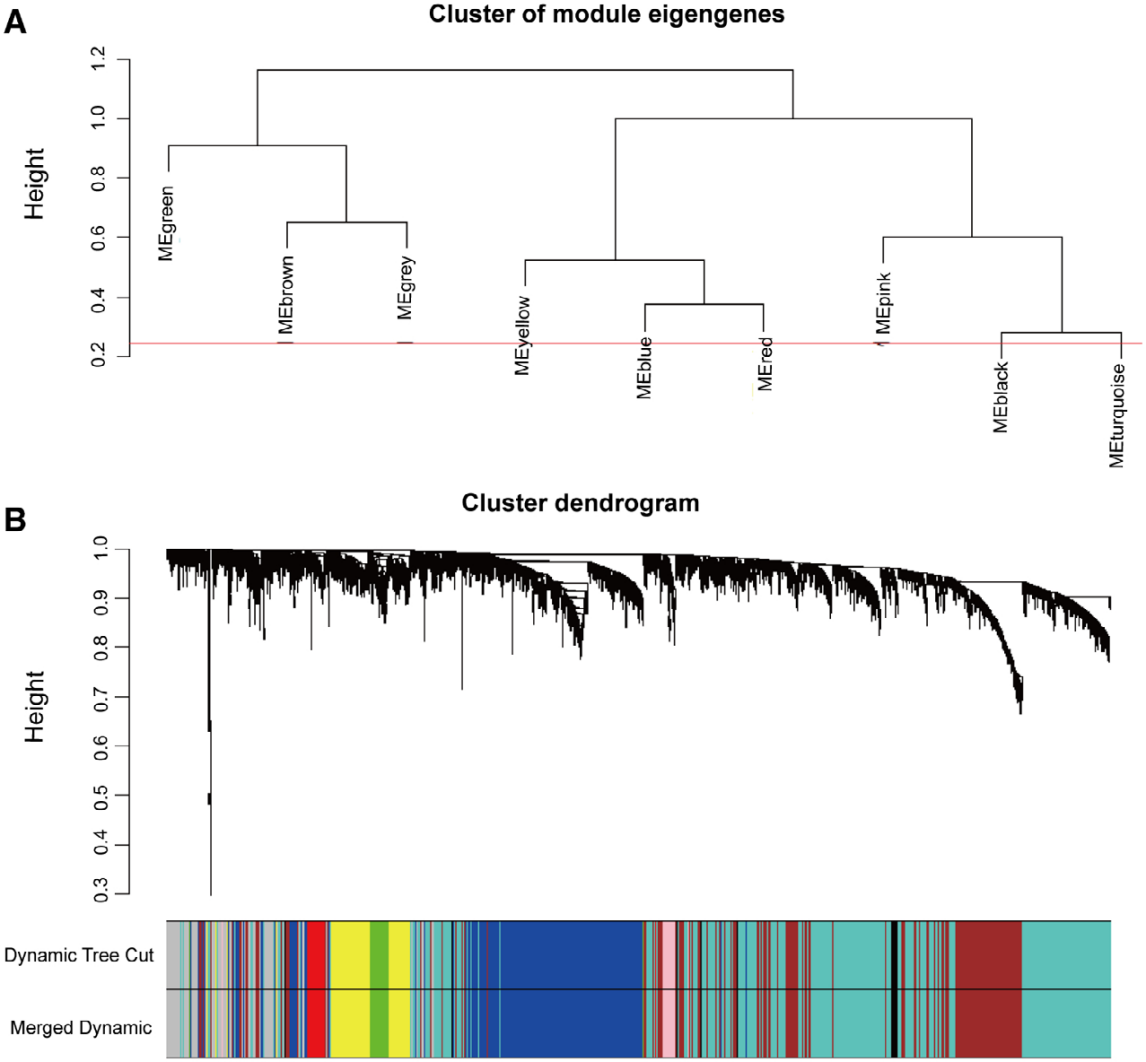
Cluster of module eigengenes and cluster dendrogram. (A) Cluster dendrogram of module eigengenes. (B) The cluster dendrogram of genes in GSE50161. Each branch of the dendrogram represents a gene. Each designated colour represents a co‐expression module.

We analysed 5000 genes, which were divided into two clusters (Fig. 1A). Our fitting degree of the scale‐free topological model was 0.85. Thus, this network conformed to the power‐law distribution and was close to the real biological network state. Network topology for thresholding powers from 1 to 20 was performed, and the relatively balanced scale independence (Fig. 1B) and mean connectivity (Fig. 1C) of the WGCNA were identified subsequently. It showed that 8 had the best power value. Thus, β = 8 was selected to produce a hierarchical clustering tree (dendrogram) of 5000 genes. As a result, nine modules were identified (Fig. 2A). Different colours represented different modules, and genes that could not be classified in any module were put into the grey module, which was removed in the subsequent analysis (Fig. 2B).

### Correlation between modules and identification of key modules

**Fig. 3:**
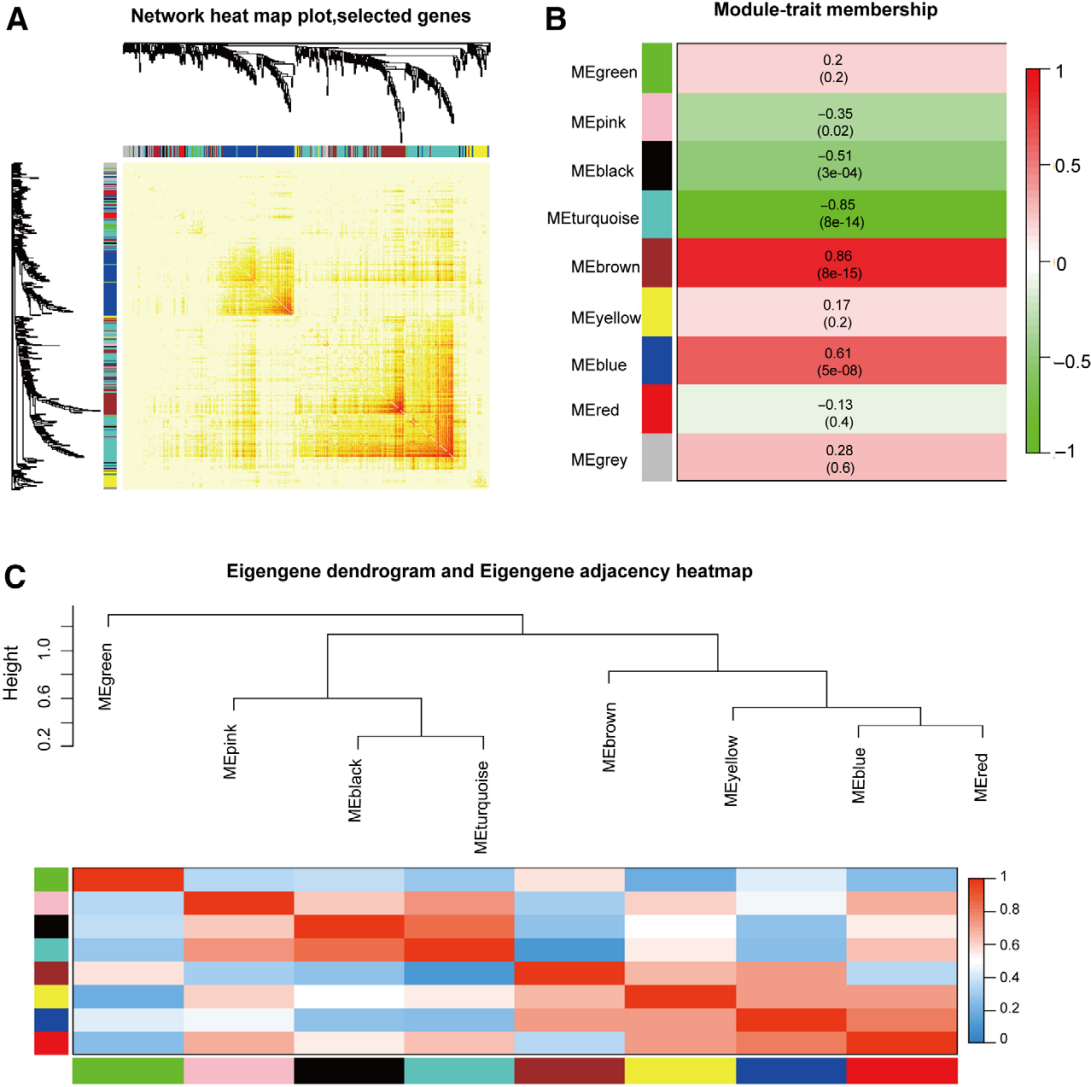
Identification of key modules. (A) Network heatmap plot of co‐expression genes. Different colours of the horizontal axis and the vertical axis represent different modules. The progressively saturated yellow and red colours indicate the high co‐expressed interrelation in the heatmap. (B) Heatmap of the correlation between module eigengenes and GBM. Each row represents a module eigengene and each column represents trait. The table is coloured by correlation according to the colour legend. The turquoise module is the most negatively correlated with GBM, and the brown module is the most positively correlated with GBM. (C) The hierarchical clustering dendrogram and the adjacency heatmap of each module. The top is the hierarchical clustering of the module crucial genes (labelled by colours). The bottom is the adjacency heatmap, where each column and row correspond to one module crucial gene (labelled by colour) or trait labelled by *y*. Red represents high adjacency (positive correlation), while blue colour represents low adjacency (negative correlation). Squares of red colour along the diagonal are the meta‐module.

**Fig. 4:**
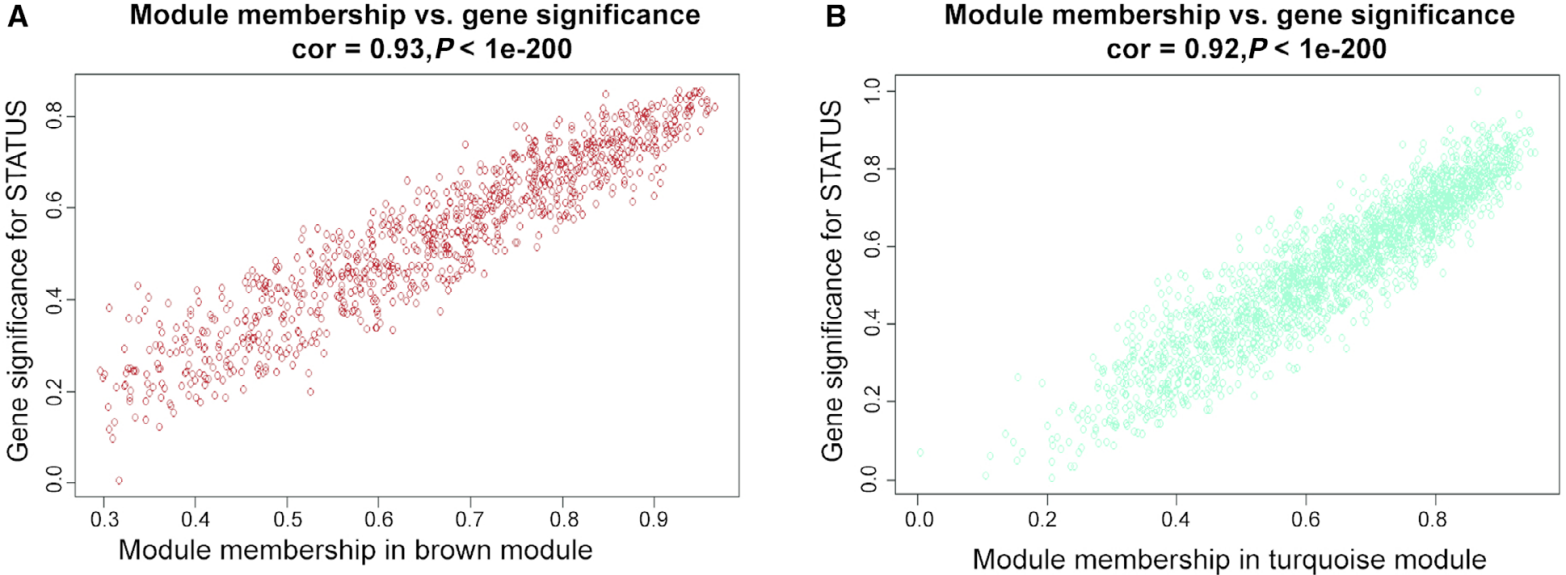
Module eigengenes in key modules. (A) Scatter plot of module eigengenes in the brown module. (B) Scatter plot of module eigengenes in the turquoise module.

The analytic results of the interaction relationships of nine modules showed that genes within modules displayed more topological overlap than the genes across modules, according to the topological overlap heatmap in the gene network (Fig. 3A). This revealed that each module was independent of each other. Similar results were demonstrated by the heatmap plotted according to adjacencies (Fig. 3C). The module eigengene of the turquoise and brown modules revealed a high correlation with disease status compared with other modules (Fig. 3B). The turquoise module (correlation index: −0.85, *P* = 8e^−14^) was negatively correlated with the disease, while the brown module (correlation index: 0.88, *P* = 8e^−15^) was positively correlated. Thus, we identified turquoise and brown modules as the key modules. Figure 4A,B illustrate the strong correlation between module membership and gene significance in brown and turquoise modules, respectively.

### Functional enrichment analysis of the two key modules

**Fig. 5:**
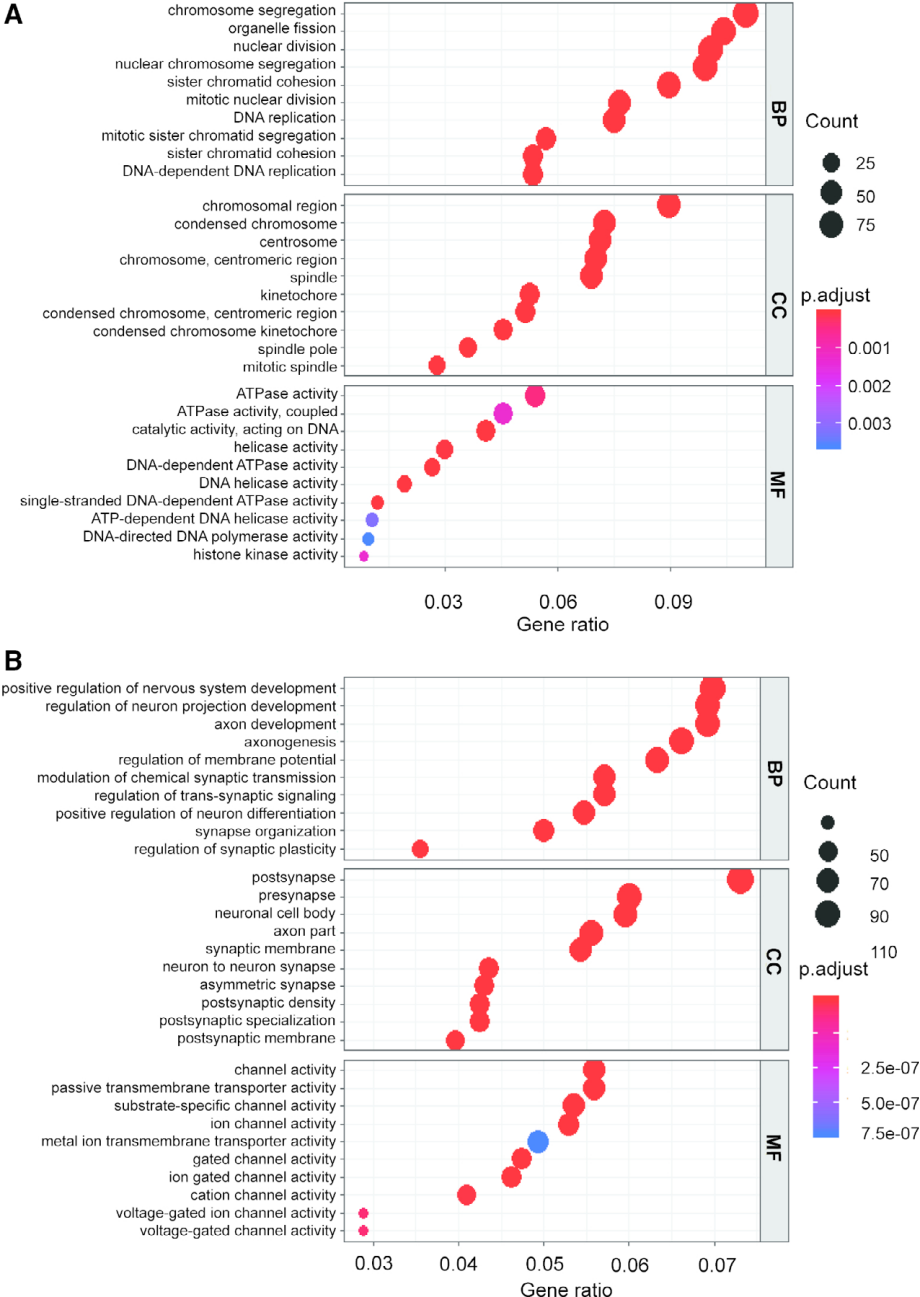
GO enrichment analysis of key modules. (A) GO enrichment analysis of genes in the brown module (top 10 in BP, CC, and MF are listed). The *y*‐axis depicts names of terms in BP, CC, and MF, respectively, and the *x*‐axis depicts gene ratio in the module. The circle size represents the count and colours represent the *P*‐value; (B) GO enrichment analysis of turquoise module genes (top 10 in BP, CC and MF are listed). CC, cellular component; MF, molecular function.

**Fig. 6:**
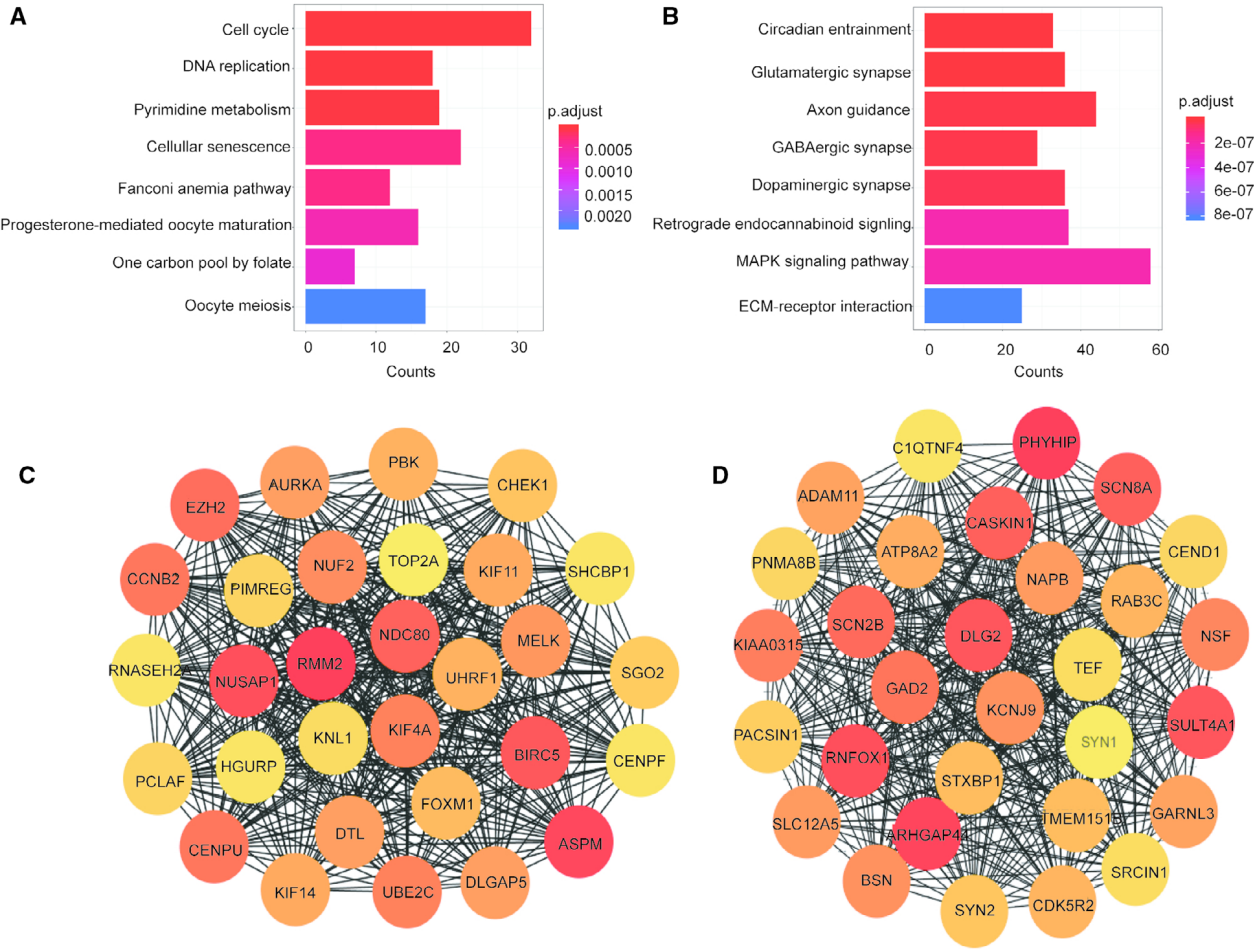
KEGG pathway enrichment and top 30 genes of key modules. (A) The KEGG pathways of the brown module. The *y*‐axis depicts the names of the terms of the pathways, and the *x*‐axis represents the count. Colours represent *P*‐values. (B) The KEGG pathways of the turquoise module. (C) In the brown module, genes with node degrees in the top 30 are displayed. (D) In the turquoise module, genes with node degrees in the top 30 are displayed. The higher the rank of the genes, the deeper the colour of the genes.

For the GO enrichment analysis of key modules, the detailed information of the brown module is shown in Fig. 5A. They were mainly enriched in cell division, including chromosome segregation, organelle fission, and nuclear division, all of which were positively correlated with tumorigenesis. Detailed information of the turquoise module is shown in Fig. 5B. They were mainly enriched in the regulation of the nervous system, such as positive regulation of neuronic development and differentiation, which were negatively correlated with tumorigenesis. Detailed information of the KEGG pathway analysis for key modules is shown in Fig. 6A,B. The brown and turquoise modules were mainly focussed on the cell cycle and neural signalling pathway, respectively. To provide solid insights, data set GSE4290 was analysed, and the results of DEGs in the data set are shown in Fig. S1A,B. KEGG pathway analysis and GO enrichment analysis of the DEGs were carried out (Figs S1C and S2). As we have seen, the KEGG pathway is enriched in pathways such as the cell cycle and GABAergic synapse. In the GO functional analysis of DEGs, the BP components of upregulated genes were mainly enriched in chromosome segregation and DNA replication, and the BP components of down‐regulated genes were mainly enriched in neuronic development and synaptic function. This finding is consistent with the functional analysis of our key modules.

### Identification of hub genes in key modules

**Fig. 7:**
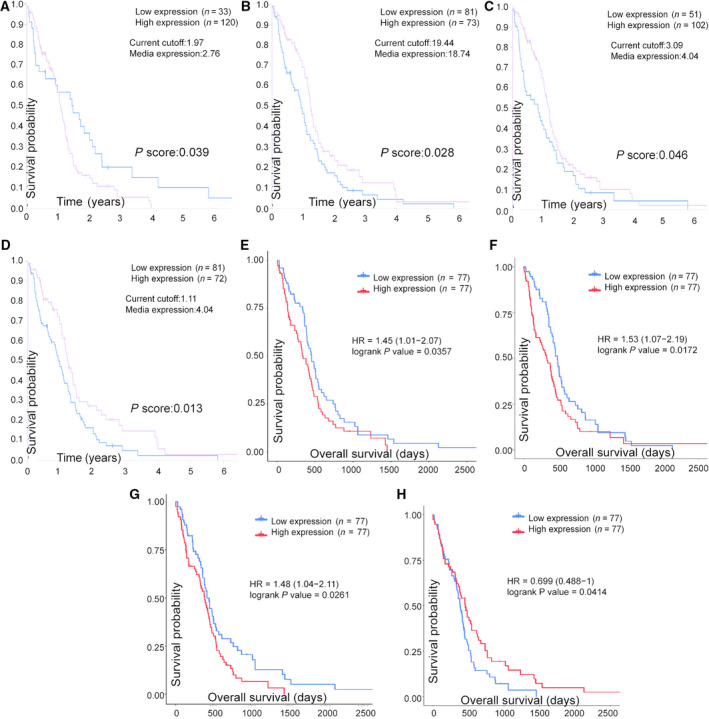
Survival analysis of hub genes. (A–D) In the brown module, genes with top 30 node degrees and significant results of survival analysis in HPA are *SHCBP1*, *NUSAP1*, *NUF2* and *KNL1* (*P* < 0.05 was regarded as significant). (E–H) In the turquoise module, genes with top 30 node degrees and significant results of survival analysis in TCGA are *SULT4A1*, *SLC12A5*, *NAPB* and *GARNL3* (*P* < 0.05 was regarded as significant).

**Fig. 8:**
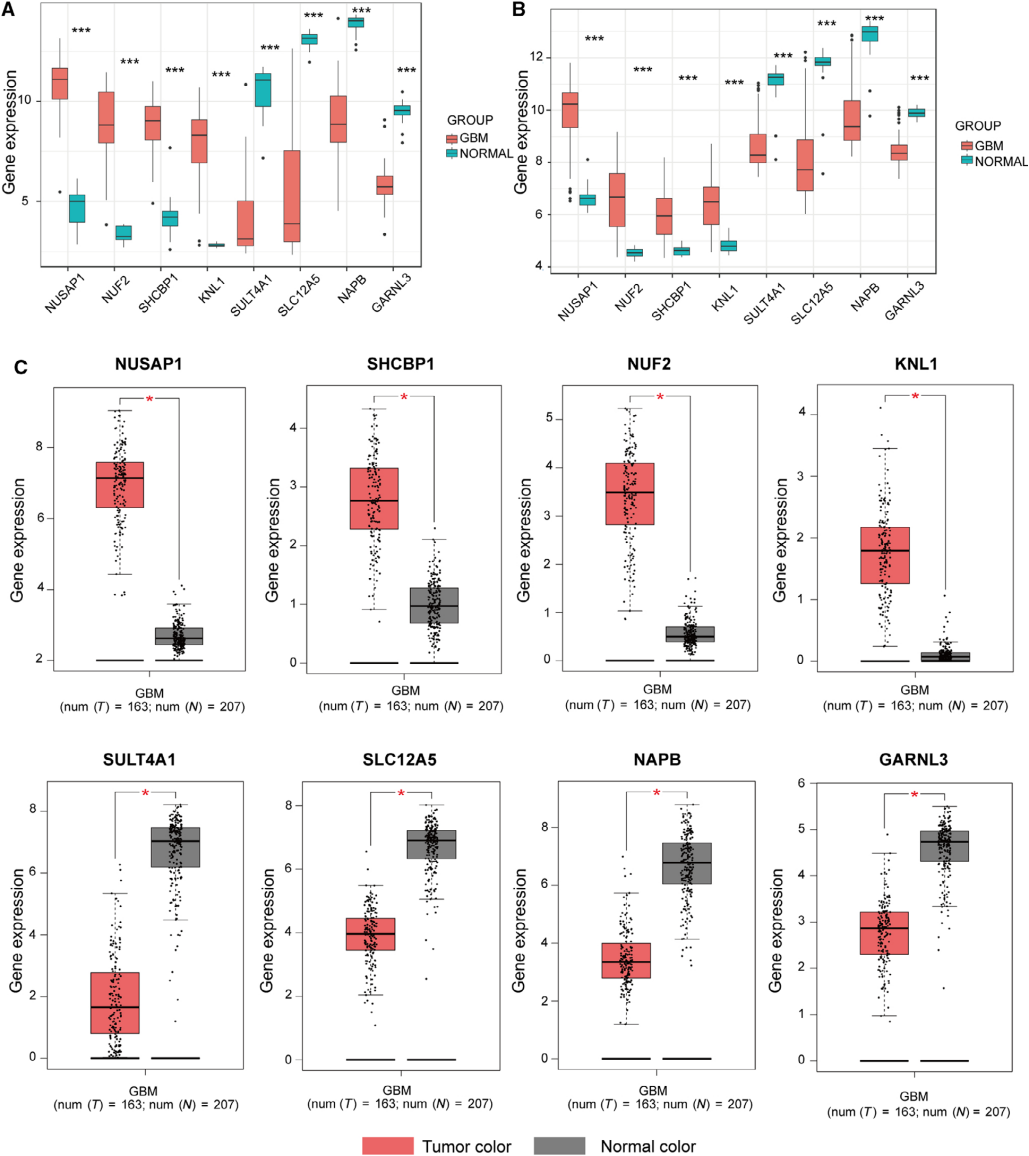
Screening and validation of hub genes at the transcriptional level. (A) Screening hub genes in GSE50161 (including 34 paediatric GBM samples and 13 normal samples). The expression status of four hub genes (*NUSAP1*, *NUF2*, *SHCBP1* and *KNL1*) was positively correlated with disease status. This was also consistent with the results for the brown module of WGCNA. The expression status of four hub genes (*SULT4A1*, *SLC12A5*, *NAPB* and *GARNL3*) was negatively correlated with disease status. This was consistent with the results in the turquoise module of WGCNA. (B) Validation of eight hub genes in GSE4290 (including 81 paediatric GBM samples and 23 normal samples), and the results were the same as earlier. (C) The differential expression levels of hub genes were demonstrated in TCGA by GEPIA. These results were consistent with the above results. These results fully demonstrated the reliability of our findings. (*t*‐test; *P < 0.05; **P < 0.01; ***P < 0.001).

The top 30 genes screened in each module using cytoscape are shown in Fig. 6C,D. Figure 7 shows the results of the survival analysis. Figure 8A is the result of the differential expression level analysis. *NUSAP1*, *SHCBP1*, *NUF2* and *KNL1* were the hub genes in the brown module and *SULT4A1*, *SLC12A5*, *NAPB* and *GARNL3* in the turquoise module.

### Validation of hub genes

**Fig. 9:**
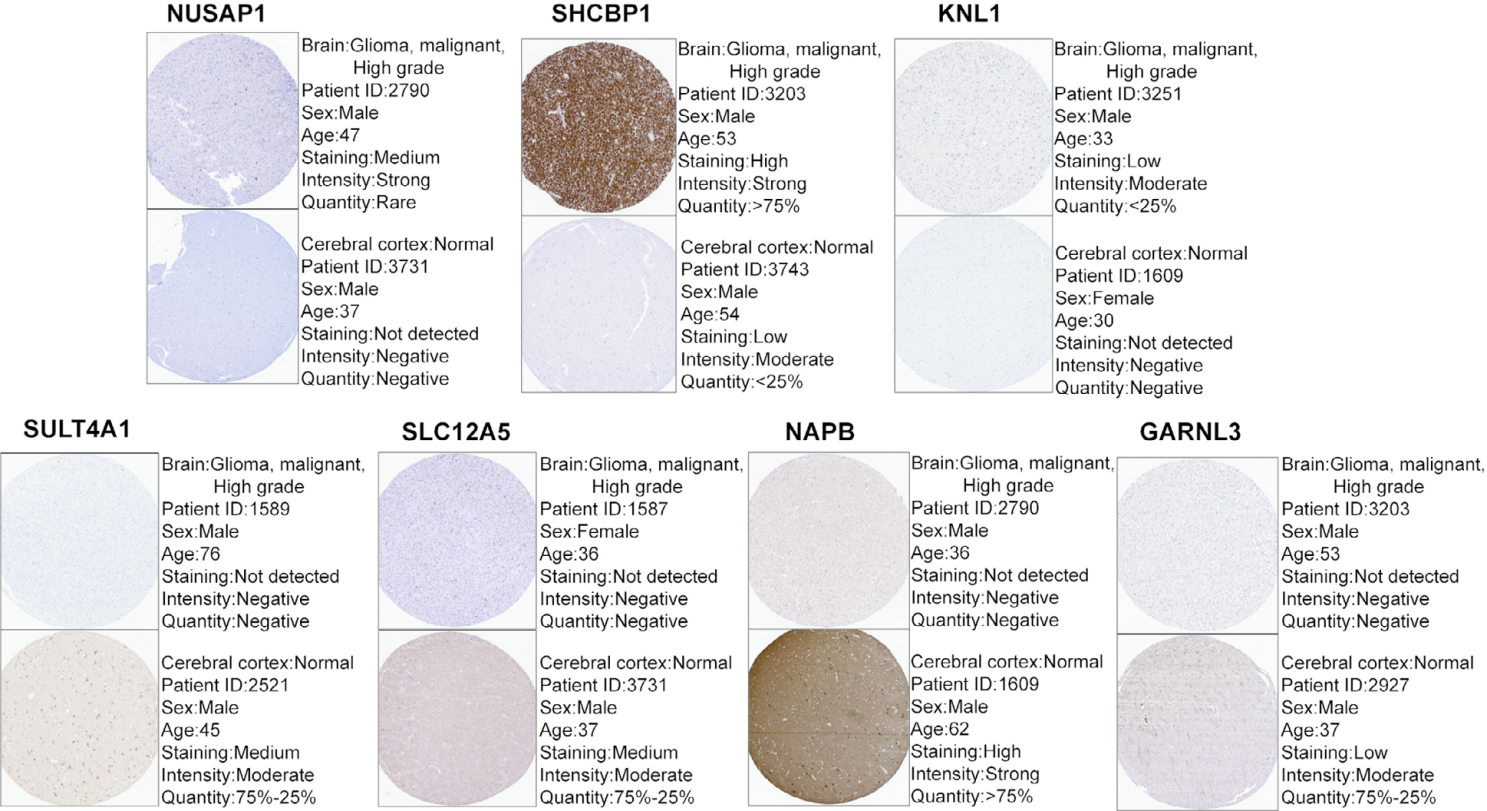
Validation of hub genes at the translational level by The HPA database. The translational expression levels of *NUSAP1*, *SHCBP1* and *KNL1* in GBM were higher than those in normal tissue. The translational expression levels of *SULT4A1*, *SLC12A5*, *NAPB* and *GARNL3* in GBM were lower than those in normal tissue.

The expression status of eight hub genes in normal and GBM samples of the other two data sets are shown in Fig. 8B,C, respectively. The results were similar to those of the previous data set. We found differences in IHC staining between the tumour samples and the normal cerebral cortex in the HPA database. It showed the translation expression levels of *NUSAP1*, *SHCBP1* and *KNL1*, which were positively correlated with disease status as they were upregulated in GBM samples. It also showed the translational expression levels of *SULT4A1*, *SLC12A5*, *NAPB* and *GARNL3*, which were negatively correlated with disease status as they were downregulated in GBM samples (Fig. 9). Unfortunately, there were no related IHC samples of *NUF2* in the database. Overall, these results showed that protein levels were consistent with previously described transcription levels.

### Hub gene expression distribution (WHO classification)

**Fig. 10:**
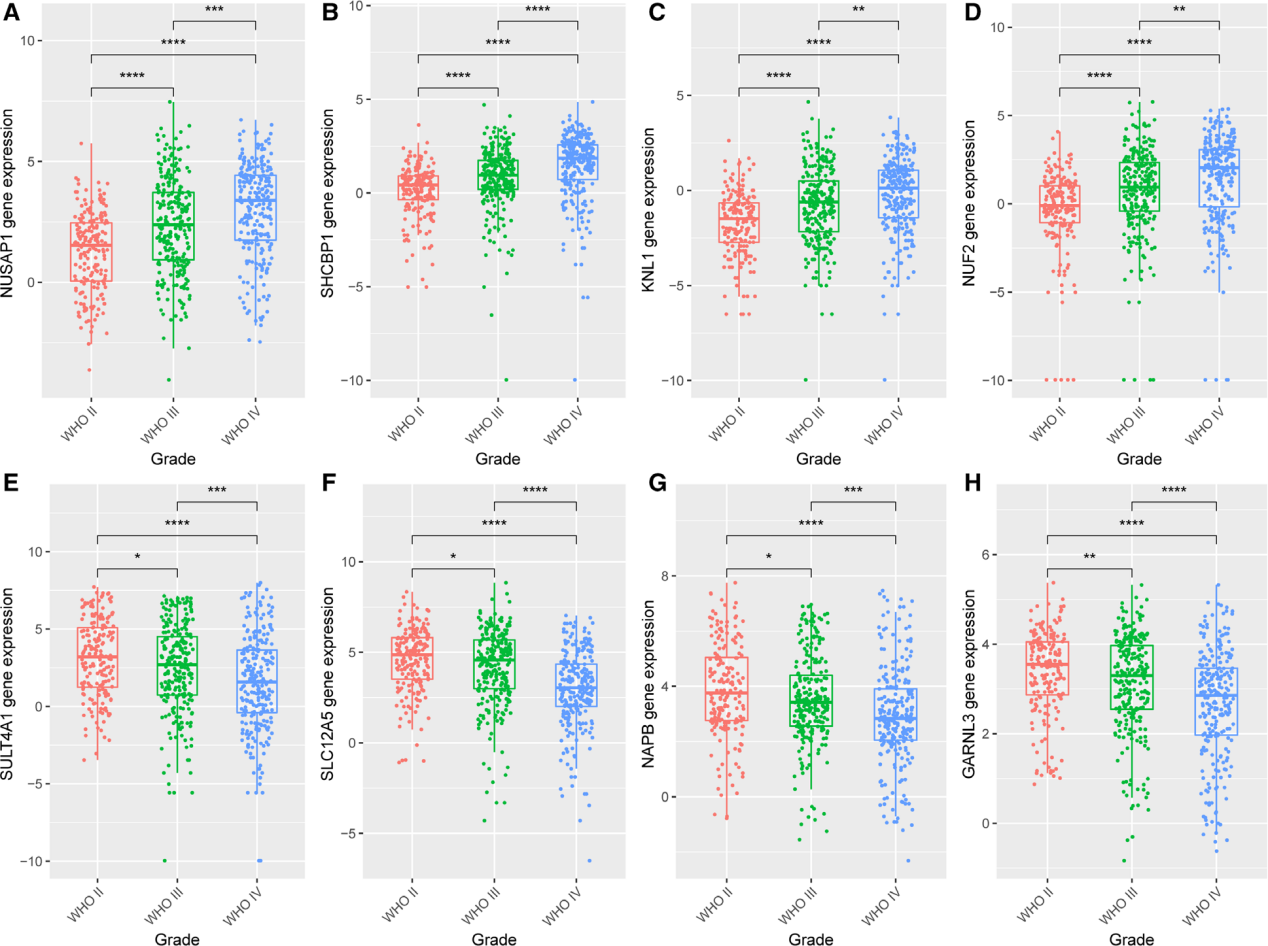
Hub gene expression distribution in the World Health Organization classification (188 GBM samples in WHO grade II, 255 samples in grade III and 249 samples in grade IV). The expression levels of *NUSAP1, SHCBP1, KNL1* and *NUF2* genes of the brown module and *SULT4A1, SLC12A5, NAPB* and *GARNL3* genes of the turquoise module were significantly different in the different World Health Organization classification. (*t*‐test; *P < 0.05; **P < 0.01; ***P < 0.001).

The expression levels of these eight hub genes, *NUSAP1, SHCBP1, NUF2, KNL1, SULT4A1, SLC12A5, NAPB* and *GARNL3*, showed significant differences in the WHO classification (Fig. 10). A higher WHO classification indicated higher expression levels of *NUSAP1, NUF2, SHCBP1* and *KNL1* genes in the brown module, and lower expression levels of *SULT4A1, SLC12A5, NAPB* and *GARNL3* in the turquoise module; the difference was statistically significant.

### TFs of hub genes

Table 2 shows the prediction of the top 20 TFs of hub genes in the brown and turquoise modules. The number of targets (hub genes) and the number of motifs/tracks for each TF are also listed in the table. E2F4 is the most important TF of *NUSAP1, SHCBP1, NUF2* and *KNL1* in the brown module, and ATF2 is the most important TF of *SULT4A1*, *SLC12A5*, *NAPB* and *GARNL3* in the turquoise module.

**Table 2:** The top 20 TFs of hub genes in key modules. NES, normalised enrichment score.

Hub genes source	TFs	NES	Targets	Motifs/Tracks
Brown module	E2F4	10.219	4	7
	SIN3A	8.997	4	7
	CRX	7.359	2	17
	PAX4	6.075	3	8
	SOX14	5.985	3	3
	ZFY	5.955	3	6
	ALX4	5.107	2	5
	FOXM1	5.032	3	4
	FOXJ2	4.927	2	9
	GTF3C2	4.672	1	7
	KLF5	4.619	2	15
	ZBED1	4.619	2	3
	ZNF423	4.589	1	1
	RXRA	4.574	2	10
	ARID3C	4.567	2	6
	ARID3A	4.544	1	1
	YY1	4.529	1	15
	NF1	4.522	1	3
	E2F3	4.507	1	3
	E1V4	4.499	1	3
Turquoise module	ATF2	6.734	4	44
	REST	6.460	3	11
	ESR1	5.375	4	4
	EBF1	4.895	3	4
	CREB1	4.885	3	5
	HOXB4	4.838	3	3
	OSR1	4.806	2	3
	TEAD4	4.668	2	1
	CLK1	4.574	2	1
	RFX2	4.369	2	30
	GCM1	4.058	3	4
	HOXA5	3.990	3	25
	PRRX1	3.916	2	4
	DBP	3.911	2	2
	ZBTB18	3.742	2	2
	NFKB1	3.516	2	1
	FOXN4	3.155	1	6
	CRX	3.110	1	1
	GATA5	3.089	1	4
	TEAD1	3.073	3	4

### The role of high expression of Hub gene about the proliferation and clone formation of GBM cells

**Fig. 11:**
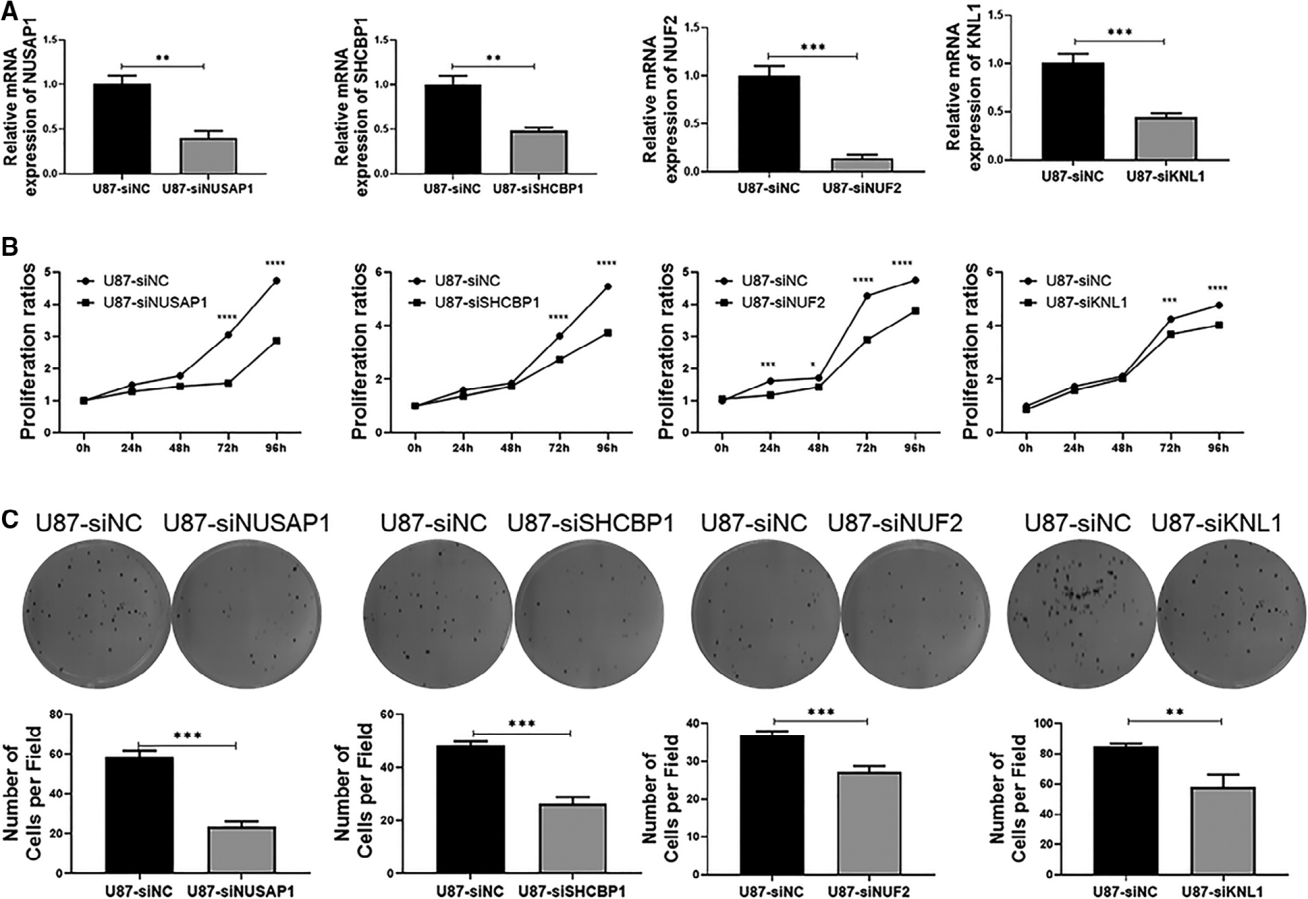
Knockout *NUSAP1, SHCBP1, NUF2* and *KNL1* gene can decrease the proliferation and cloning of U87 cells. (A) The level of *NUSAP1, SHCBP1, NUF2* and *KNL1* mRNA was determined by RT‐PCR after siRNA transfection. (B) The proliferation ability of U87 cells was evaluated by CCK8 method. (C) Clone formation analysis was used to evaluate the cloning ability of U87 cells (unpaired *t*‐test, *N* = 3, mean ± SD, *P < 0.05, **P < 0.01, ***P < 0.001 and ****P < 0.0001).

Cell proliferation and clone formation may be key events in promoting the development of cancer. Therefore, we can analyse the function of these four genes in GBM by analysing the effects of these four genes on the key events of these cancer cells. As shown in Fig. 11A, the results showed that the expression of target genes in siRNA group decreased significantly compared with siNC. We can continue to use these siRNA for functional experimental research. First, we detected the effect of knockdown target gene in the U87 cells by CCK8 method, the results as shown in Fig. 11B that the cell proliferation rate of siRNA group (siNUSAP1, siSHCBP1, siNUF2 and siKNL1) decreased significantly compared with siNC; in other words, the genes of *NUSAP1, SHCBP1, NUF2* and *KNL1* could promote cell proliferation. We further used clone formation experiment to assay the variation of proliferation and cloning ability of U87 cells. As shown in Fig. 11C, the number of clones in the siRNA group (siNUSAP1, siSHCBP1, siNUF2 and siKNL1) decreased significantly. The above results indicated that *NUSAP1, SHCBP1, NUF2* and *KNL1* play a key role in promoting the proliferation of GBM, which further confirms the reliability of our previous analysis data.

## Discussion

GBM is the most malignant glioma [27]. An in‐depth study on the molecular level and mechanism level of GBM is helpful to find new prevention and treatment targets for GBM. We used WGCNA to explore the tumorigenic factors of GBM and validated the signalling pathways and differential gene levels in two other independent data sets. Finally, two modules (brown module and turquoise module) and eight hub genes (*NUSAP1, NUF2, SHCBP1, KNL1, SULT4A1, SLC12A5, NAPB* and *GARNL3*) in the occurrence of GBM were obtained.

It has been reported that chromosomal structural variation and gene mutations are key factors influencing the occurrence and development of GBM [28, 29]. Chromosomal instability (CIN) is one of the characteristics of tumours, and nonmultiple chromosomes cause genomic alterations in cells, resulting in the acquisition of tumour characteristics in normal cells [30]. Interestingly, we found that the BP component of the GO enrichment analysis in the brown module was primarily enriched in chromosome segregation, while the cell component was mainly enriched in the spindle and chromosome, suggesting that chromosomal instability plays an active role in the occurrence of GBM. Consistent with our findings, previous studies demonstrated that the cell cycle process and chromosome instability play an important role in the tumorigenesis of GBM [31, 32]. Simultaneously, the KEGG pathway analysis of the genes in the brown module showed a consistent result. As a malignant tumour in the central nervous system, GBM is characterised by cell cycle disruption and malignant cell proliferation [33], which is in accordance with our results.

In the turquoise module, genes are mainly enriched in positive neuron development and synaptic function in the GO function annotation. The genes are mainly negatively correlated with the status of GBM, which is consistent with the progressive regulation of neurons and synapses to inhibit tumour development. Correspondingly, the main KEGG pathways in the turquoise module‐demonstrated enrichment included circadian rhythm entrainment, glutamatergic synapses, axonal guidance, GABAergic synapses, dopaminergic synapses, retrograde endogenous cannabinoid signalling, MAPK signalling pathway and extracellular matrix (ECM)–receptor interaction. Changes in circadian rhythm parameters in the mouse model were found to be associated with glioma diagnosis [34]. Netrin‐1, an axonal guidance molecule, has been reported to be associated with invasive and angiogenic phenotypes [35]. The MAPK signalling pathway regulates the normal cell cycle [36], and the ECM–receptor interaction is essential for regulating cell adhesion and cell differentiation [37]. Thus, an abnormal MAPK signalling pathway and an aberrant ECM–receptor interaction may promote GBM cell proliferation, prevent cell differentiation and increase its invasiveness.

As a pivotal stage of the cell cycle, DNA replication and chromosome segregation are thought to play critical roles in tumorigenesis. The dynamics of microtubule defects and spindle anomalies lead to CIN, which produces a multilayer genomic instability that is common in human cancers [38]. During cell division, the spindle assembly checkpoint (SAC) prevents the separation of repeated chromosomes until each chromosome is properly attached to the spindle device [39]. Error‐free chromosome separation relies on a stable connection between the kinetochore and spindle microtubules, ensuring the correct separation of chromosomes during cell division [40]. Interestingly, the four hub genes (*NUSAP1*, *NUF2*, *SHCBP1* and *KNL1*) in the brown module are associated with microtubules and spindles. *NUSAP1* encodes nucleolar and spindle‐associated protein 1, a nucleolar spindle‐associated protein that plays a role in spindle microtubule organisation [41]. *NUF2* encodes cell division associated 1, responsible for kinetochore–microtubule attachment under normal physiological conditions, and is, therefore, an essential protein for the isolation of sister chromatids during mitosis [42]. *SHCBP1* encodes Src homologous and collagen (SHC) SH2‐binding protein 1, a protein essential for midbody organisation and cytokinesis completion [43]. *KNL1* encodes kinetochore scaffold 1, which is involved in microtubule attachment to chromosome centromeres and the activation of spindle checkpoints during mitosis [44]. Many studies have reported that some of the four hub genes in the brown module are cancer‐associated genes that play a positive role in tumorigenesis and malignant phenotype in glioma. *NUSAP1* is a prognostic factor for gliomas [45], and silencing *NUSAP1* can inhibit GBM cell proliferation both *in vivo* and *in vitro* [46]. Knockdown of *NUF2* by small interfering RNA can inhibit tumour growth and induce apoptosis in human glioma cells [47]. *SHCBP1* is highly expressed in gliomas and promotes proliferation and invasion of glioma cells by activating the NF‐κB signalling pathway [48]. *KNL1* is a type of cancer/testis antigen [49] and has been confirmed to be highly expressed in high‐grade glioma cell lines and glioma patients, but its detailed function in glioma has not been explored [50]. We predicted the TFs of four hub genes, and some of them are correlated with gliomas in previous reports. For example, E2F4 is potentially a key transcriptional regulator in GBM that regulates the transcription of multiple genes [51]. KLF5 is involved in GBM angiogenesis by regulating angiogenic factors with G‐patch and FHA domain1 (*AGGF1*) expression [52]. FOXM1 activates *MMP2* to enhance the invasiveness of gliomas [53]. In summary, all four hub genes in the brown module are associated with microtubules and spindles, and chromosome separation error is vital in tumorigenesis. It should be noted that *NUF2* participates in the normal function of SAC as part of the nuclear division cycle 80 (NDC80) complex, while *KNL1* is directly involved in the activation of SAC. Meanwhile, *NUSAP1* and *KNL1* directly or indirectly influence the repair of DNA damage [54, 55]. Based on our results, we speculate that the abnormally high expression of the four hub genes leads to CIN or DNA repair disorders during the cell division process and normal cells acquire cancer cell characteristics.

We also screened *SULT4A1*, *SLC12A5*, *NAPB* and *GARNL3* in the turquoise module that was downregulated in data sets and TCGA. *SULT4A1* encodes sulfotransferase family 4A member 1, a brain‐specific sulfotransferase involved in the metabolism of neurotransmitters [56]. *SLC12A5* encodes a potassium and chloride transporter of the SLC12 family, which is exclusively expressed in the central nervous system and retina [57]. *NAPB* encodes the NSF attachment protein beta, which is preferentially expressed in brain tissues [58]. *GARNL3* encodes a GTPase‐activating Rap/RanGAP domain like 3, associated with the positive regulation of GTPase activity. These four hub genes are preferentially expressed in brain tissues, and their dysfunction can lead to neuropsychiatric diseases. For example, *SULT4A1* and *SLC12A5* are associated with schizophrenia [59, 60]. Alternatively, spliced isoforms of *NAPB* are associated with autism, and *GARNL3* is linked with intellectual disability [61, 62]. There are few studies on the three genes (*SULT4A1*, *NAPB*, *GARNL3*) in GBM, and a few studies have reported the possibility of *SLC12A5* as a biomarker for GBM [63]. However, due to the significant differences in their expression in GBM, further research is needed. For the TF prediction of these genes, we found that some TFs demonstrated a positive regulation of nervous system development and function in previous reports. For example, defects in ATF‐2 cause dysplasia and neurological abnormalities in mice [64]. REST is involved in coordinating the neural induction and differentiation processes [65]. ESR1 polymorphisms are associated with the risk of developing dementia [66]. In summary, all four hub genes are associated with the normal development of the nervous system and can be considered negatively associated with tumorigenesis. The proteins encoded by the above four hub genes play a key role in energy metabolism, which is vital for normal cellular processes, especially in the central nervous system. Therefore, further research into the role and mechanism of these genes in the development of GBM is necessary.

In addition, this study also found that these eight hub genes all had significant differences in the WHO grading of glioma. The expression levels of these genes in the WHO classification were consistent with the results of our study, further revealing the potential of these genes as biomarkers. In general, the above results provide further insights that enhance our understanding of the pathogenesis of GBM at the molecular level.

This study has several limitations. First, it focussed on bioinformatics data mining and analysis. Further mechanistic studies need to be performed to understand the detailed role of these genes in GBM fully. Second, due to the limitations in data availability, this study did not perform a comprehensive analysis of GBM subtypes based on clinical data. Finally, we validated our findings only in a single Gene Expression Omnibus data set and TCGA database, and use of more data sources would be required for further verification.

## Conclusions

This study explored the tumorigenic factors of GBM using WGCNA. We identified two modules (brown and turquoise) and eight hub genes (*NUSAP1*, *NUF2*, *SHCBP1*, *KNL1*, *SULT4A1*, *SLC12A5*, *NAPB* and *GARNL3*) in the occurrence of GBM. The brown module plays a positive role in GBM tumorigenesis, primarily in the cell cycle, chromosome separation and DNA replication. Simultaneously, the turquoise module plays a negative role in tumorigenesis, primarily in the positive regulation of nervous system development and cell differentiation. These two key modules enhance our understanding of tumorigenic mechanisms in patients with glioblastoma. In addition, these eight hub genes and corresponding TFs may act as prognostic biomarkers and therapeutic targets for GBM.

## Conflict of interest

The authors declare no conflict of interest.

## Author contributions

CL conceived and designed the experiments, analysed the data, prepared figures and/or tables, authored or reviewed drafts of the paper, approved the final draft. BP conceived and designed the experiments, authored or reviewed drafts of the paper. LG analysed the data and contributed materials /analysis tools. MZ conceived and designed the experiments, approved the final draft. HS performed the experiments. YY performed the experiments. LL conceived and designed the experiments, and approved the final draft.

## Supporting information


**Fig. S1.** Visualizing DEGs in GSE4290 and KEGG pathway enrichment. Note: (A) The number of genes that had an absolute fold change greater than 1.5 and a P‐value less than 0.05. (B) Heat map hierarchical clustering showed that the top 100 DEGs in GBM groups compared with control groups. (C) KEGG pathway enrichment analysis of the DEGs in GSE4290 (P‐value less than 0.001 are shown). Up‐regulated pathways are labelled red, and down‐regulated pathways are labelled blue. Abbreviations: DEGs, different expression genes; KEGG, Kyoto Encyclopedia of Genes and Genomes.

**Fig. S2.** GO enrichment analysis of DEGs in GSE4290. Note: GO enrichment analysis of up‐regulated genes; (B) GO enrichment analysis of down‐regulated genes.. Top 10 in BP, CC and MF are listed. Abbreviations: GO, gene ontology; BP, biological process; CC, cellular component; MF, molecular function; ATP, adenosine triphosphate.

## Data Availability

The following information was supplied regarding data availability: All the original data in this study were downloaded from the public databases including GEO (https://www.ncbi.nlm.nih.gov/geo/) and TCGA (https://portal.gdc.cancer.gov/). For the hub genes identification, we used GSE50161 (https://www.ncbi.nlm.nih.gov/geo/query/acc.cgi?acc=GSE50161), GSE4290 (https://www.ncbi.nlm.nih.gov/geo/query/acc.cgi?acc=GSE4290) and GEPIA (http://gepia.cancer‐pku.cn/). The translational expression level data sets were from the HPA database (https://www.proteinatlas.org/). As the prediction of TFs, we used the iRegulon plugin (http://iregulon.aertslab.org/) in cytoscape. The resource of gene expression distribution level was from the CGGA database (http://www.cgga.org.cn/).
