# Supplementary figures and images for: Identification of key modules and hub genes in glioblastoma multiforme based on co‐expression network analysis
Source: FEBS Open Bio. 2021 Feb 9;11(3):833–50. doi: 10.1002/2211-5463.13078 (PMC7931238; doi:10.1002/2211-5463.13078)

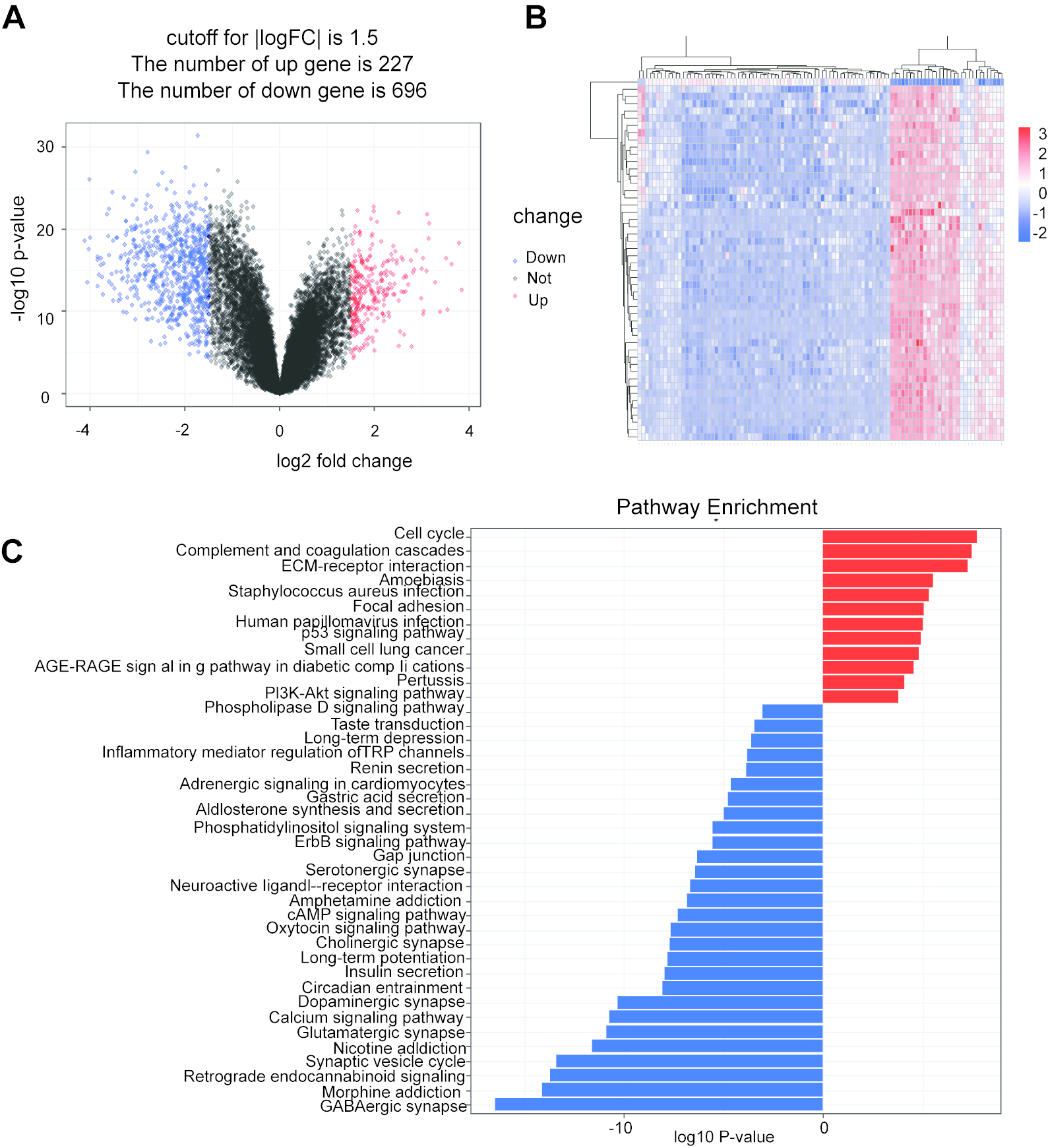

Supplement: Supplementary file 1 — Fig. S1. Visualizing DEGs in GSE4290 and KEGG pathway enrichment. Note: (A) The number of genes that had an absolute fold change greater than 1.5 and a P‐value less than 0.05. (B) Heat map hierarchical clustering showed that the top 100 DEGs in GBM groups compared with control groups. (C) KEGG pathway enrichment analysis of the DEGs in GSE4290 (P‐value less than 0.001 are shown). Up‐regulated pathways are labelled red, and down‐regulated pathways are labelled blue. Abbreviations: DEGs, different expression genes; KEGG, Kyoto Encyclopedia of Genes and Genomes. [file FEB4-11-833-s001.jpg]

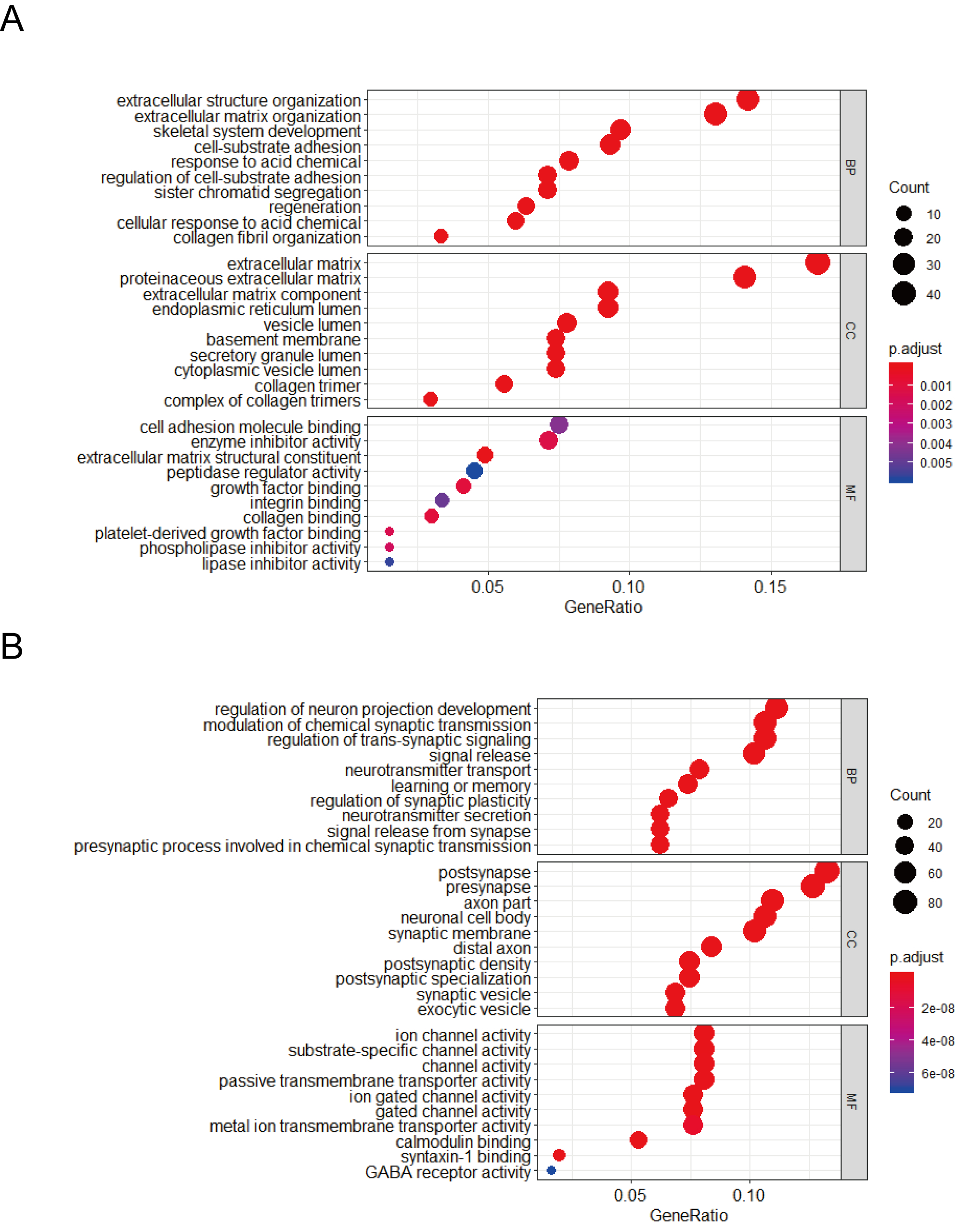

Supplement: Supplementary file 2 — Fig. S2. GO enrichment analysis of DEGs in GSE4290. Note: GO enrichment analysis of up‐regulated genes; (B) GO enrichment analysis of down‐regulated genes.. Top 10 in BP, CC and MF are listed. Abbreviations: GO, gene ontology; BP, biological process; CC, cellular component; MF, molecular function; ATP, adenosine triphosphate. [file FEB4-11-833-s002.jpg]
